# Carbon monoxide and nitric oxide diffusion capacity of formerly exposed asbestos workers with high-resolution computed tomography in a cross-sectional study

**DOI:** 10.1038/s41598-025-99824-w

**Published:** 2025-05-02

**Authors:** Julia Krabbe, Damian J. Reimers, Nelly Otte, Panagiotis Doukas, Timm Dirrichs, Thomas Radtke, Holger Dressel, Thomas Kraus

**Affiliations:** 1 Institute of Occupational, Social and Environmental Medicine, Medical Faculty, RWTH Aachen, Pauwelsstraße 30, 52074 Aachen, Germany; 2 Department of Vascular Surgery, Medical Faculty, European Vascular Centre Aachen-Maastricht, RWTH Aachen University, Aachen, Germany; 3 Department of Diagnostic and Interventional Radiology, Medical Faculty, RWTH Aachen University, Aachen, Germany; 4 Epidemiology, Biostatistics and Prevention Institute (EBPI), Division of Occupational and Environmental Medicine, University of Zurich, Zurich, Switzerland; 5 Department of Internal Medicine, Division of Occupational and Environmental Medicine, University Hospital Zurich, Zurich, Switzerland

**Keywords:** DLNO, DLCO, Asbestosis, Pleural plaque, Pleural thickening, Respiratory tract diseases, Diagnosis

## Abstract

Asbestos exposure can induce pulmonary fibrosis known as asbestosis, and pleural thickening, as well as various cancers. Although lung diffusion capacity (DL) including nitric oxide (NO) is assumed to be more sensitive than carbon monoxide (CO), its added value in assessment of pneumoconiosis has not been investigated yet. 371 formerly exposed workers visiting the outpatient clinic for assessment including pulmonary function testing with DL and low-dose high resolution computed tomography between 2018 and 2021 were included. Subgroups were formed depending on findings in low-dose high resolution computed tomography classified according to ICOERD. Receiver operating characteristic curve (ROC) analysis revealed some diagnostic accuracy for DLNO (AUC = 0.73; 95% confidence interval 0.64–0.82) and DLCO (AUC = 0.70; 95% confidence interval 0.60–0.79) regarding asbestosis, but not per unit alveolar volume. DLCO and DLNO correlated strongly with a decreasing score of irregular opacities according to ICOERD (*ρ*_DLCO_ = − 0.87, *ρ*_DLNO_ = − 0.85) but DLNO was also susceptible to emphysema. Although tendencies of a more sensitive detection of diffusion capacity impairment were observed, DLNO was not clearly superior to DLCO in assessment of asbestosis. Based on our findings and considering the lack of availability of DLNO in clinical routine, DLNO does not seem to have added value for clinical assessment of formerly asbestos exposed workers. Future studies should further investigate DLNO including healthy controls and confounders such as emphysema and smoking.

## Introduction

Asbestos exposure is one of the most frequent causes of pneumoconiosis in Germany^1^ and worldwide^2^. Asbestos was used for decades before its ban 1993 in Germany and 2005 in the EU, but due to the latency of up to 60 years between exposure and manifestation of asbestos-associated diseases corresponding cases still occur^3^. Asbestos exposure is associated with benign and malignant diseases. Cancer of lungs, larynx, ovaries and malignant mesothelioma are official occupational diseases in Germany^4^. Benign conditions include asbestosis and pleural thickening. Asbestosis is an asbestos induced pulmonary fibrosis and typically presents as bilateral usual interstitial pneumonia (UIP) pattern with apicobasal gradient with basal and peripheral subpleural predominance^5,6^. However, some studies also describe other patterns such as nonspecific interstitial pneumonia (NSIP)^7^. Asbestos associated pleural findings include pleural thickening of pleura parietalis and visceralis, as well as mediastinal wall and diaphragm and can exhibit partial or full calcification^8,9^. For standardized assessment of asbestos related changes the International Classification of Occupational and Environmental Respiratory Diseases (ICOERD) is used and bilateral irregular and/or linear opacities in lower lung fields are indicative of asbestosis^9–11^. However, if no accompanying pleural thickening are found, fibrotic lesions cannot be attributed to asbestosis easily^9,12^.

In Germany it is mandatory to offer persons with former asbestos exposure periodical surveillance examinations even after cessation of exposure^13–16^. Similarly, patients with acknowledged occupational diseases are offered follow-up examinations of their pneumoconioses. Those appointments include physical examination, low-dose high resolution computed tomography or X-ray of the chest, pulmonary function testing and lung diffusion capacity (DL). While pleural thickening only cause slight reductions in vital capacity and/or total lung capacity^17^, asbestosis can lead to relevant restrictive ventilation disorders, diffusion impairment and disruption of gas exchange possibly ending in hypoxemia, need of oxygen therapy and death^18^.

DLCO has traditionally been used to assess diffusion impairment in interstitial lung diseases, including asbestosis. However, it reflects both alveolar-capillary membrane conductance (Dm) and pulmonary capillary blood volume (Vc)^19^, which could limit its sensitivity in detecting specifically early fibrotic changes. In asbestosis, initial pathological changes predominantly affect the alveolar-capillary membrane, while capillary blood volume may remain relatively preserved. This could result in a normal or only slightly reduced DLCO despite early disease. DLNO in contrast, is assumed to be more specific to Dm and largely independent of capillary blood volume^20^, making it potentially a more sensitive tool for detecting early interstitial alterations. Furthermore, the dependency of diffusion of CO on haemoglobin concentration with relevant reductions in diffusion capacity in case of anemia requires a determination of current haemoglobin concentration with consecutive correction^21^. DL including nitric oxide (NO) is independent of haemoglobin concentration^19^. If DLNO provides a clinically relevant advantage to detect impairment in diffusion capacity due to pneumoconiosis has not been investigated yet.

In this study pulmonary function testing with spirometry, DLCO and DLNO were conducted in formerly asbestos exposed workers enrolled in surveillance programs or visits for assessment of their occupational disease who simultaneously received a low-dose high resolution computed tomography. Depending on conditions determined in low-dose high resolution computed tomography, changes in DL and pulmonary function testing were compared. Based on the assumption that DLNO is more sensitive to conduction changes of the alveolar capillary membrane than DLCO the hypothesis was that asbestos-related alveolar cell and basal membrane thickening could be detected earlier in DLNO than DLCO.

## Results

### Characteristics of included patients

371 male patients were included in this study. Mean age was 70.7 years ± 7.45 (SD) with a range between 53 and 85 years.

The characteristics of patients are listed in Table 1 for overall and subgroups. The subgroup ‘pleural findings’ (97 patients) included 64 patients with only parietal type of pleural abnormalities, 7 patients with only visceral type of pleural abnormalities and 26 patients with both types. ‘Mixed findings’ (113 patients) contained 71 patients (62.8% of mixed findings) with pleural findings and emphysema, 6 patients (5.3%) with irregular opacities and emphysema and 36 patients (31.9%) with pleural findings, irregular opacities, and emphysema.

## Pulmonary function testing

Overall, patients presented with FVC, FEV1 and FEV1/FVC above lower limit of normal (Table 2). In subgroups, FVC was significantly reduced in patients with pleural findings, asbestosis with a score of irregular opacities of at least 4 and mixed findings compared to patients with no findings. FVC as percentage of lower limit of normal (LLN) (FVC%LLN) was not significantly reduced. FEV1 was reduced for patients with asbestosis (score at least 4), emphysema and mixed findings, while FEV1 as percentage of LLN (FEV1%LLN) and FEV1/FVC were only reduced for patients with emphysema.

**Table 1:** Anthropometric data, preexisting conditions and medication of study patients. Percentages are based on the number of available data for each group.

	Overall	Groups					
		No findings	Only pleural findings	Asbestosis score 2–3 with and without pleural findings	Asbestosis score ≥ 4 with and without pleural findings	Only emphysema	Mixed findings
	*n* = 371	*n* = 82	*n* = 97	*n* = 17	*n* = 22	*n* = 40	*n* = 113
	Median (range)						
Age (years)	71 (53–85)	65 (55–84)	72 (56–85)	71 (59–80)	76.5 (60–85)	66 (54–81)	72 (53–85)
Height (m)	1.74 (1.56–1.98)	1.77 (1.62–1.98)	1.74 (1.56–1.95)	1.77 (1.69–1.89)	1.73 (1.61–1.89)	1.76 (1.61–1.94)	1.73 (1.6–1.89)
Weight (kg)	89 (56–151)	93 (59–141)	85 (64–136)	91 (79–103)	88 (64–122)	88 (58–128)	86 (56–151)
Body Mass Index (BMI)	29 (19.4–48.4)	30.1 (21.6–41.5)	28.2 (22.1–39.7)	29.6 (25.9–31.9)	28.9 (24.3–40.1)	28.7 (19.4–43.5)	28.5 (21.1–48.4)
*Smoking (n = 353)*							
Never smokers [n (%)]	80 (23)	19 (25)	24 (26)	5 (29)	5 (23)	7 (18)	20 (19)
Active smokers [n (%)]	59 (17)	16 (21)	13 (14)	2 (12)	1 (5)	15 (38)	12 (11)
Former smokers [n (%)]	214 (61)	42 (54)	55 (60)	10 (59)	16 (72)	17 (44)	74 (70)
Pack years [median (range)]	29 (0–180)	35 (0–156)	15.5 (0–96)	39 (0–97)	26 (0–180)	42 (0–140)	25.5 (0–140)
*Occupational exposure*							
Asbestos exposure duration (years) [median (range)] (*n* = 330)	20 (0.1–151)	15 (0.2–40)	25 (1–58.6)	22 (6–151)	29 (5–41)	13.5 (0.1–44)	22 (2.9–46)
Cumulative asbestos exposure (fibre years) [median (range)] (*n* = 192)	21 (0.1–755)	18 (0.1–501.3)	26 (1.3–754.6)	27.8 (10.5–308.6)	27.5 (9–116.2)	20 (0.1–332.2)	25.9 (0.1–523.7)
History of (*n* = 303)*	n (%)						
COPD	55 (18)	7 (22)	14 (23)	2 (13)	2 (12)	9 (28)	21 (26)
Asthma bronchiale	11 (4)	1 (3)	3 (5)	1 (6)	1 (6)	2 (6)	3 (4)
Pulmonary hypertension	0 (0)	0 (0)	0 (0)	0 (0)	0 (0)	0 (0)	0 (0)
Cardiac insufficiency	9 (3)	1 (3)	2 (3)	0 (0)	0 (0)	4 (13)	2 (2)
Congestive heart disease	88 (29)	11 (34)	20 (33)	6 (38)	9 (53)	11 (34)	31 (38)
Heart valve disease	14 (5)	2 (6)	3 (5)	2 (13)	1 (6)	1 (3)	5 (6)
Diabetes mellitus	62 (20)	10 (32)	19 (31)	5 (30)	4 (23)	5 (16)	19 (24)

**Table 2:** Pulmonary function for all patients and for subgroups. Data are presented as mean ± sd, p-value for comparison to ‘no findings’.

Pulmonary function testing	Overall *n* = 371	Groups										
		No findings *n* = 82	Only pleural findings *n* = 97		Asbestosis score 2–3 with and without pleural findings *n* = 17		Asbestosis score ≥ 4 with and without pleural findings *n* = 22		Only emphysema *n* = 40		Mixed findings *n* = 113	
	Mean (SD)	Mean (SD)	Mean (SD)	p-value	Mean (SD)	p-value	Mean (SD)	p-value	Mean (SD)	p-value	Mean (SD)	p-value
FVC [l]	3.88 (0.84)	4.21 (0.72)	3.84 (0.90)	0.01	4.01 (0.94)	0.85	3.34 (0.87)	< 0.001	3.9 (0.75)	0.19	3.75 (0.79)	0.001
FVC [%LLN]	128.55 (23.20)	130.04 (17.65)	131.81 (25.23)	0.92	131.36 (31.93)	0.92	121.34 (31.93)	0.39	121.45 (20.24)	0.25	128.16 (23.17)	0.92
FVC [%pred]	95.15 (16.87)	97.39 (12.64)	97.00 (18.52)	0.99	97.11 (19.36)	0.99	88.67 (22.98)	0.13	91.00 (14.93)	0.20	94.39 (16.75)	0.66
FEV1 [l]	2.83 (0.71)	3.12 (0.66)	2.85 (0.7)	0.05	2.93 (0.75)	0.79	2.58 (0.68)	0.006	2.64 (0.77)	0.002	2.69 (0.67)	0.001
FEV1 [%LLN]	126.89 (30.18)	128.63 (23.45)	132.42 (31.54)	0.87	130.5 (28.94)	0.97	128.81 (34.58)	0.98	109.89 (29.4)	0.006	125.97 (31.29)	0.90
FEV1 [%pred)	91.57 (20.51)	94.27 (16.27)	95.49 (20.11)	0.99	93.73 (20.69)	0.99	90.98 (23.79)	0.95	80.67 (21.53)	0.003	89.94 (21.51)	0.48
FEV1/FVC	0.73 (0.09)	0.73 (0.07)	0.75 (0.08)	0.93	0.73 (0.07)	0.99	0.78 (0.07)	0.23	0.70 (0.11)	< 0.001	0.72 (0.09)	0.34
FEV1/FVC [%LLN]	116.04 (15.96)	115.75 (11.72)	120.47 (13.47)	0.11	116.69 (11.93)	0.99	126.22 (12.30)	0.0098	104.81 (16.78)	< 0.001	115.42 (15.53)	0.99
FEV1/FVC [%pred]	95.35 (12.66)	96.21 (9.42)	98.44 (10.56)	0.59	95.67 (9.34)	0.99	102.53 (9.65)	0.08	87.19 (14.14)	< 0.001	94.38 (12.16)	0.73

## Lung diffusion capacity (DL) – DLCO and DLNO

**Fig. 1:**
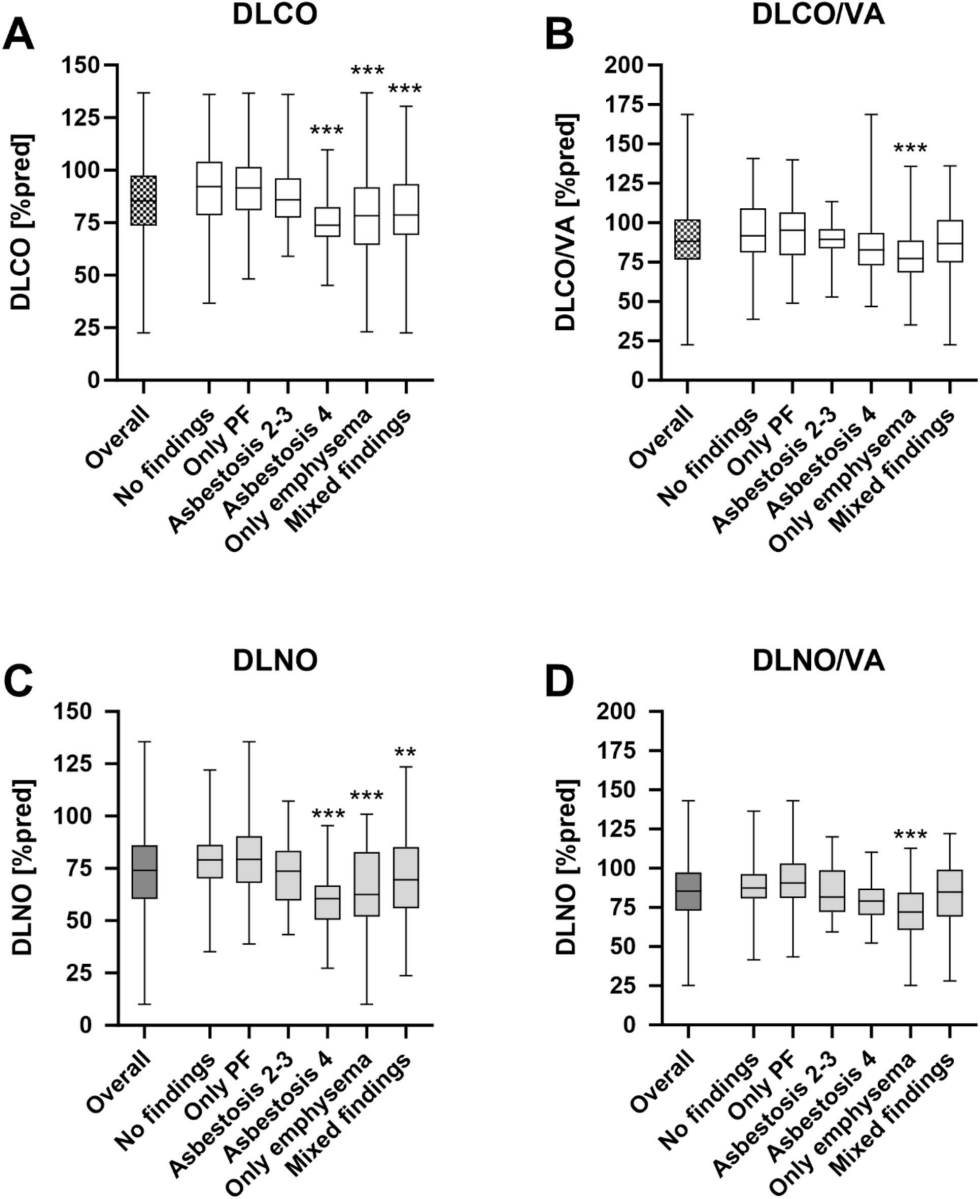
Diffusion capacity testing (DCT) for all patients and for subgroups. (**A**) DLCO, (**B**) DLCO/VA, (**C**) DLNO and (**D**) DLNO/VA for all patients (*n* = 371) and subgroups. Box plots with median and min to max, ** = *p* < 0.01 and *** = *p* < 0.001 for comparison to ‘no findings’.

For subgroups, a reduced DLCO was detectable for patients with asbestosis (score at least 4), emphysema and mixed findings compared to patients with no findings (Table 3; Fig. 1A). For DLCO/VA differences could only be detected for patients with emphysema and mixed findings (Table 3) and for DLCO/VA %pred only for emphysema (Table 3; Fig. 1B). Regarding DLNO, DLNO %pred and DLNO/VA reductions were observed for patients with asbestosis (score at least 4), emphysema and mixed findings (Table 3; Fig. 1C), while DLNO/VA %pred was reduced for patients with emphysema and mixed findings (Table 3; Fig. 1D). Alveolar volume (VA) was reduced for pleural findings, asbestosis (score at least 4) and mixed findings (Table 3) and VA %pred for asbestosis (score at least 4) and mixed findings (Table 3).

For identification of associations of DLCO [%pred] and DLNO [%pred] and smoking with adjustment for asbestos exposure (fibre years) and CT findings (sum grade of irregular opacities, emphysema and pleural changes according to ICOERD), multiple linear regressions were performed (Table 4). Asbestos exposure and asbestos associated morphological changes of the lungs and pleura were included in the model due to reported associations with reduction in DL^21,22^. For DLCO and DLNO, significant relationships were found for extend of asbestosis and emphysema but not for pack years, cumulative asbestos exposure (fibre years) or pleural thickening. For every increase in one point of score for irregular opacities DLCO and DLNO decreased 0.02%pred and for every increase in one point of score for emphysema DLCO decreased 0.02%pred and DLNO 0.14%pred, respectively.

**Table 3:** Lung diffusion capacity (DL) – DLCO and DLNO - for all patients and for subgroups. Data are presented as mean ± sd, p-value for comparison to ‘no findings’.

DL	Overall *n* = 371	Groups										
		No findings *n* = 82	Only pleural findings *n* = 97		Asbestosis score 2–3 with and without pleural findings *n* = 17		Asbestosis score ≥ 4 with and without pleural findings *n* = 22		Only emphysema *n* = 40		Mixed findings *n* = 113	
	Mean (SD)	Mean (SD)	Mean (SD)	p-value	Mean (SD)	p-value	Mean (SD)	p-value	Mean (SD)	p-value	Mean (SD)	p-value
DLCO [mmol min^− 1^ kPa^− 1^]	7.46 (1.93)	8.44 (1.96)	7.74 (1.71)	0.05	7.75 (1.81)	0.52	6.04 (1.27)	< 0.001	7.10 (0.34)	< 0.001	6.86 (1.74)	< 0.001
DLCO [%LLN]	115.76 (26.03)	122.72 (25.18)	124.17 (22.82)	0.99	119.04 (24.00)	0.98	102.70 (18.50)	0.004	104.76 (29.62)	0.001	109.42 (25.93)	0.001
DLCO [%pred]	85.14 (19.11)	90.98 (18.47)	90.92 (16.80)	0.98	87.34 (17.42)	0.70	74.85 (13.34)	< 0.001	77.63 (21.91)	< 0.001	80.27 (18.83)	< 0.001
DLCO/VA [mmol min^− 1^ kPa^− 1^]	1.22 (0.28)	1.29 (0.28)	1.27 (0.25)	1.00	1.22 (0.19)	0.82	1.16 (0.32)	0.21	1.08 (0.29)	< 0.001	1.16 (0.27)	0.01
DLCO/VA [%LLN]	122.98 (27.97)	127.60 (28.07)	130.51 (24.42)	0.94	124.50 (21.91)	0.99	120.19 (33.44)	0.72	106.65 (27.54)	< 0.001	119.26 (28.15)	0.15
DLCO/VA [%pred]	89.07 (20.22)	93.25 (20.34)	94.14 (17.73)	1.00	89.96 (15.36)	0.97	86.27 (23.94)	0.48	78.00 (20.30)	< 0.001	86.02 (20.17)	0.05
DLNO [mmol min^− 1^ kPa^− 1^]	27.33 (8.20)	32.00 (6.98)	28.32 (7.59)	0.007	27.14 (6.83)	0.08	20.20 (5.50)	< 0.001	25.64 (9.32)	< 0.001	25.10 (7.87)	< 0.001
DLNO [%LLN]	104.43 (29.81)	109.45 (24.28)	114.49 (27.46)	0.70	102.52 (25.24)	0.86	89.10 (25.06)	0.02	87.29 (29.19)	< 0.001	101.51 (33.15)	0.23
DLNO [%pred]	73.02 (19.81)	79.11 (15.71)	78.81 (18.35)	0.92	72.34 (17.36)	0.33	59.56 (15.10)	< 0.001	63.32 (21.17)	< 0.001	69.79 (21.23)	0.002
DLNO/VA [mmol min^− 1^ kPa^− 1^]	15.46 (3.35)	16.87 (2.98)	16.21 (3.15)	0.51	15.30 (2.53)	0.24	13.66 (2.38)	< 0.001	13.92 (3.77)	< 0.001	14.70 (3.34)	< 0.001
DLNO/VA [%LLN]	109.82 (24.91)	112.48 (21.74)	118.47 (22.65)	0.35	111.02 (25.03)	0.99	103.71 (20.25)	0.44	91.99 (23.74)	< 0.001	107.78 (26.76)	0.57
DLNO/VA [%pred]	85.03 (18.61)	88.41 (16.30)	91.19 (17.05)	0.51	85.62 (17.81)	0.56	79.07 (14.35)	0.11	72.42 (18.69)	< 0.001	82.85 (19.68)	0.002
VA [liter]	5.26 (0.95)	5.68 (0.76)	5.23 (0.86)	0.005	5.31 (0.87)	0.43	4.43 (0.93)	< 0.001	5.44 (0.99)	0.54	5.06 (1.00)	< 0.001
VA [%LLN]	105.68 (15.83)	110.69 (10.59)	107.01 (14.02)	0.40	105.48 (17.94)	0.63	93.19 (18.3)	< 0.001	107.03 (16.92)	0.65	102.88 (17.70)	0.003
VA [%pred]	85.11 (12.80)	89.30 (8.53)	86.09 (11.31)	0.32	84.92 (14.50)	0.59	74.9 (14.71)	< 0.001	86.30 (13.65)	0.65	82.79 (14.27)	0.002

**Table 4:** Multiple linear regression - Lung diffusion capacity (DL) – DLCO and DLNO. Scores for irregular opacities and emphysema according to ICOERD (sum grades for irregular opacities and emphysema respectively, between 0 and 18).

*n* = 190	*p* ANOVA	corr. *R*^2^		
DLCO [%pred]	SE	Beta	p	B (95% CI)
	< 0.001	0.14		
Smoking (Pack years)	0.0004	− 0.04	0.60	− 0.0002 (− 0.001 to 0.001)
Cumulative asbestos exposure (fibre years)	0.0001	0.10	0.14	0.0002 (− 0.00006 to 0.0004)
Score for irregular opacities (sum grade)	0.006	− 0.23	0.001	− 0.02 (− 0.03 to − 0.009)
Score for emphysema (sum grade)	0.004	− 0.27	< 0.001	− 0.02 (− 0.03 to − 0.009)
Pleural thickening (yes / no)	0.03	0.03	0.36	0.03 (− 0.03 to 0.08)

*n* = 190	*p* ANOVA	corr. *R*^2^		
DLNO [%pred]	SE	Beta	p	B (95% CI)
	< 0.001	0.16		
Smoking (Pack years)	0.0004	− 0.03	0.63	− 0.0002 (− 0.001 to 0.001)
Cumulative asbestos exposure (fibre years)	0.0001	0.13	0.06	0.0002 (− 0.000007 to 0.0004)
Score for irregular opacities (sum grade)	0.006	− 0.28	< 0.001	− 0.02 (− 0.03 to − 0.01)
Score for emphysema (sum grade)	0.004	− 0.25	< 0.001	− 0.14 (− 0.02 to − 0.007)
Pleural thickening (yes / no)	0.02	0.02	0.29	0.007 (− 0.04 to 0.05)

## Lung diffusion capacity (DL) for detecting asbestosis – ROC-curve analysis

**Table 5:** ROC-curve analysis for asbestosis (score at least 4).

	AUC (95% CI)	*p* value	Cut off (%pred)	Sensitivity (%)	Specificity (%)
DLCO	0.70 (0.60–0.79)	0.002	76.38	68.18	70.77
DLNO	0.73 (0.64–0.82)	< 0.001	65.99	77.27	67.34
DLCO/VA	0.59(0.47–0.70)	0.17	95.94	81.82	38.11
DLNO/VA	0.62 (0.51–0.72)	0.06	88.15	81.82	45.85

**Fig. 2:**
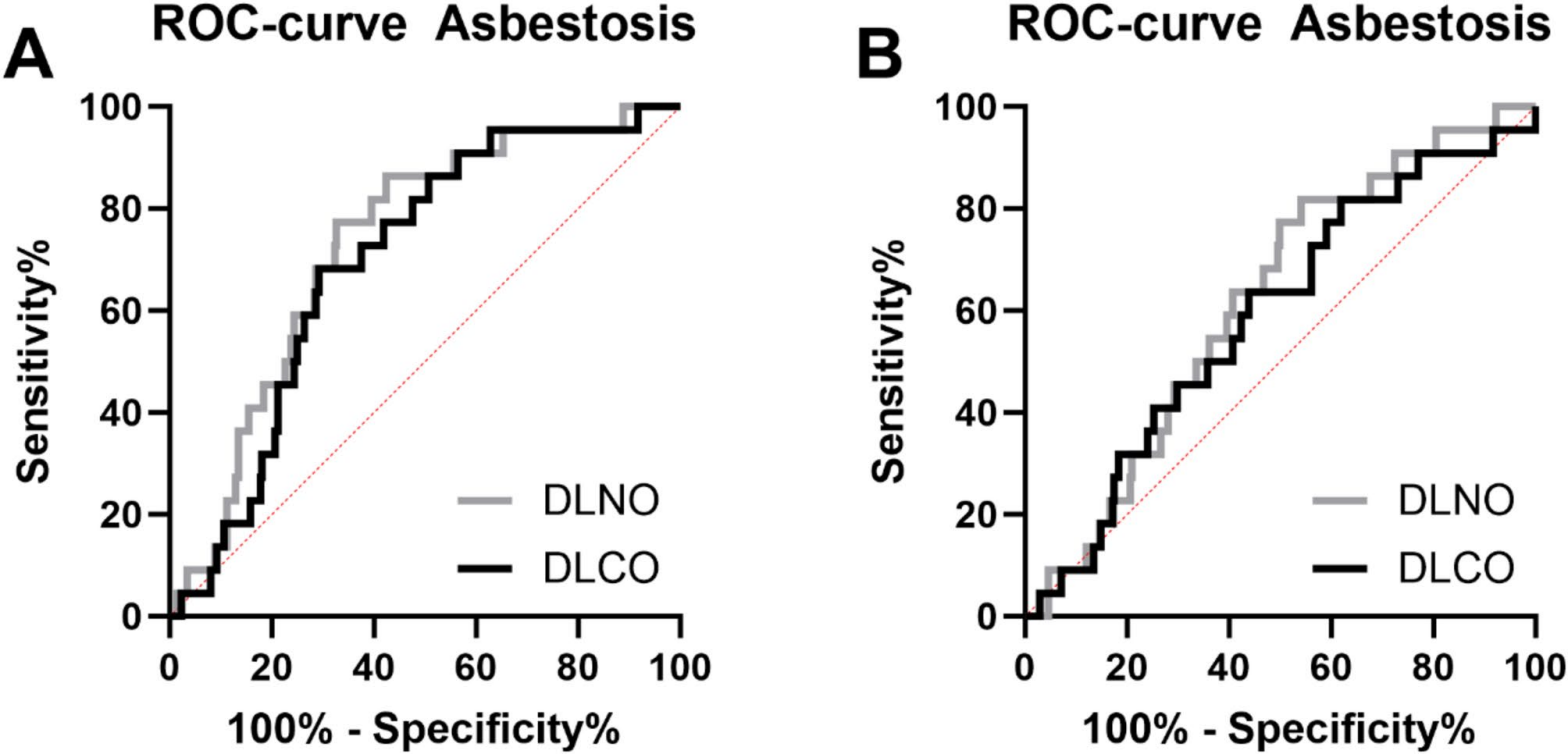
ROC-curves for detection of asbestosis (score at least 4). (**A**) DLCO & DLNO, (**B**) DLCO/VA & DLNO/VA.

In ROC-curve analysis (Fig. 2) the area under the curve (AUC) was significantly different from 0.5 for DLCO and DLNO but not DLCO/VA and DLNO/VA (Table 5). Highest AUC was achieved for DLNO with a sensitivity of 77.27% and a specificity of 67.34%.

## DL in relation to score of irregular opacities

**Fig. 3:**
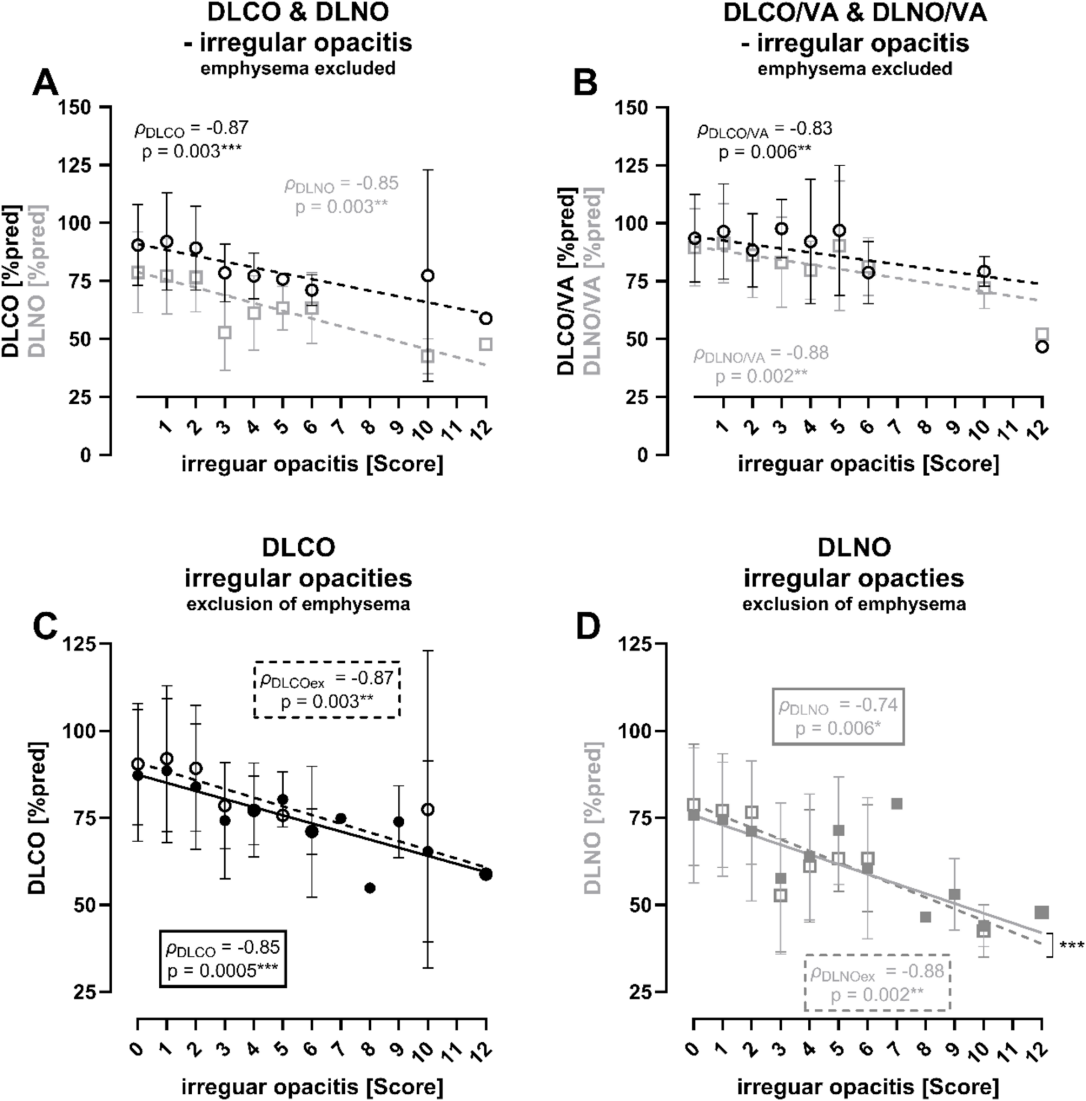
Diffusion capacity in relation to irregular opacities according to ICOERD classification ^6^. (**A**) DLCO (black) and DLNO (grey) for irregular opacities, *n* = 224, all patients with emphysema with a score of at least 1 were excluded. (**B**) DLCO/VA (black) and DLNO/VA (grey) for irregular opacities, *n* = 224, all patients with emphysema with a score of at least 1 were excluded. (**C**) DLCO before (solid line, *n* = 371) and after (dashed line, *n* = 224) exclusion of all patients with emphysema with a score of at least 1 (**D**) DLNO before (solid line, *n* = 371) and after (dashed line, *n* = 224) exclusion of all patients with emphysema with a score of at least 1. Mean ± SD plus simple linear regression with Pearson correlation.

In a subgroup of patients without emphysema, DLCO and DLNO (Fig. 3A) as well as DLCO/VA and DLNO/VA (Fig. 3B) were negatively correlated with an increasing score of irregular opacities. When comparing correlations before and after exclusion of patients with emphysema no significant differences in correlation coefficients were found for DLCO (differences of both *ρ*,* z* = 0.855, *p* = 0.196) (Fig. 3C). In contrast, we found slightly lower correlation coefficients for DLNO when patients with emphysema were excluded (*z* = 5.003, *p* < 0.001) (Fig. 3D).

## Discussion

This study is the first to report pulmonary function testing including DLCO and DLNO in individuals formerly exposed to asbestos. Patients presented pulmonary function above lower limits of normal. Reduced DLCO and DLNO were detected in asbestosis and emphysema while DLCO/VA and DLNO/VA were reduced only in emphysema. ROC-curve analysis revealed some diagnostic accuracy for DLCO and DLNO, but not DLCO/VA and DLNO/VA for detection of asbestosis, with highest AUC for DLNO. Both, DLCO and DLNO strongly correlated with decreasing score of irregular opacities according to ICOERD, but the strength of correlation did not differ between DLCO and DLNO. In contrast to DLCO, DLNO was susceptible to interference by emphysema to a similar extent.

The patient base visiting the outpatient clinic formerly exposed to asbestos presented overall no consecutive physical impairment regarding the lungs. In subgroups, no restrictive ventilation pattern was detectable but only a reduction in FVC in patients with asbestosis or pleural plaques compared to patients without findings but no general impairment. This is in accordance with other studies reporting about formerly exposed asbestos workers in Germany with comparable exposure^22,23^ and worldwide^24,25^. Likewise, DLCO/VA was not reduced except in patients with emphysema compared to the group of patients without findings. Interestingly, this cohort presented higher DLCO and DLCO/VA than comparable studies^22,23^. This could be explained with a larger number of patients with pulmonary fibrosis and emphysema in those studies. In our study, nearly half of patients presented no findings or only pleural findings in low-dose high resolution computed tomography with no impairments in pulmonary function or diffusion capacity. Furthermore, patients with asbestosis with a score of at least four according to ICOERD presented reduced FVC, DLCO, DLNO and VA but not DLCO/VA and DLNO/VA compared to the group of patients without findings. These findings stress the earlier effects of pulmonary fibrosis on diffusion capacity as reductions in lung volumes disappear when expressed per unit alveolar volume. Emphysema also had significant influence on DL but is mostly caused by cigarette smoking^26^. Accordingly, while the proportion of active smokers was comparable between the subgroups of patients with emphysema, pleural findings only and no findings, the group of patients with emphysema reported substantially higher pack years than the subgroup with pleural findings (Table 1).

Patients aware of pleural abnormalities detected on their CT scans could have demonstrated a higher likelihood of smoking cessation compared to those informed of normal CT findings^27^. Beyond its established role in causing emphysema, smoking is hypothesized to reduce diffusing capacity for carbon monoxide (DLCO) even in the absence of emphysema^28^. However, in the multivariable model adjusted for relevant confounders, cumulative smoking exposure (measured in pack-years) was not significantly associated with DLCO or DLNO in this study. In contrast, asbestosis and emphysema were significantly correlated with reductions in both DLCO and DLNO, though the effect sizes were minimal and lacked clinical relevance. Notably, the models accounted for only 14 and 16% of the variability in DLCO and DLNO respectively, indicating that unmeasured factors likely influence diffusing capacity, thereby precluding definitive conclusions from these analyses. Nevertheless, the data suggest that smoking may not have contributed to the observed reductions in diffusing capacity, although the uneven distribution of smokers and pack-years across groups warrants caution in interpretation.

Regarding asbestosis, a data-driven analysis revealed some diagnostic accuracy for DLCO and DLNO, but not DLCO/VA and DLNO/VA. However, in diagnostic medicine a high specificity is required to guarantee protection against unnecessary further possibly invasive diagnostics according to quaternary prevention^29^. Set with a specificity of at least 90% sensitivity was around 14% for DLCO and DLNO. However, in comparison, DLNO showed the highest diagnostic accuracy for asbestosis. All lung diffusion parameters (i.e., DLCO, DLNO, DLCO/VA and DLNO/VA) showed strong inverse correlations with asbestosis. Similar findings were reported for asbestos exposed workers’ low-dose and high resolution CT (HRCT)^30–33^ and radiographs classified according to ILO^34^. Accordingly, in another study DLCO/VA of formerly asbestos exposed workers without findings in CT was comparable to the data in the current study for workers without findings^35^. Thus, as recommendations already include DL should always be included for pulmonary function testing in exams of formerly asbestos-exposed individuals with a higher sensitivity for functional impairment than pulmonary function testing alone^36^.

Consequently, diffusion capacity is feasible for determination of severity of asbestosis or emphysema but did not show great diagnostic accuracy. A limitation of this study is the small number of patients with severe asbestosis. Of 22 patients with an asbestosis score of at least four more than half had an exact score of 4. Due to the ban of asbestos in Germany 30 years ago patients with a high cumulative asbestos exposure and consecutive asbestosis are more likely dead or their health status does not allow to visit the outpatient clinic regularly. Furthermore, decreased DLCO is associated with poor prognosis in asbestosis patients^36^, patients with impairment in diffusion capacity would have visited the clinic less frequently. Thus, a selection of rather healthier patients could have occurred which could be indicated by an overall DLCO slightly higher compared to other studies^22^.

Although, DLNO is independent of haemoglobin concentration and reported to be more sensitive to conduction changes of the alveolar capillary membrane than CO^20^ it has not been investigated for asbestos-associated pneumoconiosis. Generally, it is assumed that DLCO is influenced by Dm and vascular conductance assumed to be somewhat equal to pulmonary capillary blood volume (Vc), while DLNO is mostly independent of vascular conductance due to rapid reaction with haemoglobin^20^. Thus, in participants with inhalation of hypertonic saline solution^19^. DLNO was more sensitive to report impairment in Dm. Consecutively, in idiopathic pulmonary fibrosis (IPF) with usual interstitial pneumonia (UIP) and nonspecific interstitial pneumonia (NSIP) patterns, and other pulmonary fibrosis such as systemic sclerosis, DLNO is reported to reflect computed tomography-related assessment of fibrosis more sensitive than DLCO due to better reflection of Dm^37,38^. In asbestosis, the diffusion impairment primarily concerns Dm due to alveolar cell and basal membrane thickening, while idiopathic pulmonary fibrosis and other pulmonary fibrosis such as systemic sclerosis affect Dm and vascular components^39^. Thus, if DLNO is truly more independent from vascular conductance than DLCO, it should have been more sensitive to detect possible diffusion capacity impairing effects. Furthermore, in emphysema with alveolar function loss Dm is considerably decreased, and DLNO/VA seems to be more sensitive to emphysema detection than DLCO/VA^40^. Accordingly, DLNO showed a slightly higher diagnostic accuracy in ROC-curve analysis than DLCO. However, DLNO/VA and DLCO/VA showed both no significant diagnostic accuracy. Furthermore, regarding correlation of DL with score of irregular opacities, DLNO was influenced by simultaneous presence of emphysema, but DLCO was not. This stresses the higher sensitivity of DLNO regarding changes in Dm in comparison to DLCO. Similarly, in a study with patients with cystic fibrosis in which Dm appears more affected than vascular conductance and Vc, computed tomography scores correlated best with DLNO^41^. In bronchiolitis obliterans syndrome, the most common form of chronic lung allograft dysfunction, DLNO was best compared to body plethysmography to differentiate patients with no bronchiolitis obliterans syndrome early stages^42^. In this study, DLNO showed the highest diagnostic accuracy for asbestosis detection but an equal correlation to irregular opacities than DLCO. Both, DLCO and DLNO showed strong correlation with severity of asbestosis and DLNO/VA had a slightly stronger correlation than DLCO/VA. So, a tendency of higher sensitivity of DLNO could be postulated. However, no clear advantage of DLNO in comparison to DLCO could be identified. Furthermore, DLNO was considerable influenced by co-existence of emphysema while DLCO was not. Emphysema is common among formerly asbestos exposed workers and seems to be associated with asbestosis^43^, although primarily caused by corresponding smoking history^26,44^. Accordingly, in this study the subgroup with mixed findings all patients had emphysema. So overall, 152 of 371 patients, i.e., 41%, of all formerly asbestos exposed patients presented with emphysema. Since different combinations of emphysema and asbestos-related pleural or parenchymal changes are combined the mixed findings subgroup, no specific analysis or conclusion can be drawn from the results. In summary, the benefit in sensitivity of DLNO regarding Dm seems to be countervailed by susceptibility for other conditions impairing primarily Dm. To differentiate between conditions affecting Dm and Vc simultaneous DLCO and determination of Dm and Vc could be necessary. Future large sized studies are warranted with both DLCO and DLNO covering the entire disease severity spectrum of asbestosis.

The diffusion capacities of the study population were similar to other studies with formerly exposed workers^22,23^. Studies with asbestos exposed patients without abnormal findings in computed tomography and/or radiographs showed no pathological reduction in DLCO^45–48^, however, control groups of patients without abnormal findings were very low in some studies. As a limitation this study did not include healthy non-exposed controls. Thus, possible slight impairments in Dm and consecutively DL without morphological changes detectable in low-dose high resolution computed tomography could not have been identified. DLCO, DLNO, DLCO/VA and DLNO/VA were all below 100% predicted for the group of patients with no findings in high resolution computed tomography but above lower limit of normal. DLCO values observed in these patients were lower than those reported for healthy controls in previous studies^25^. Reference values for DLNO are less well-established compared to those for DLCO but even here DLNO was not below lower limit of normal. Thus, the hypothesis that asbestos exposure could have effects on diffusion capacity even before morphological changes that are detectable in low-dose high resolution computed tomography has to be investigated further. Future studies with appropriate patient numbers should include healthy controls matched for relevant confounding factors.

This cross-sectional study has limitations regarding the representativeness of the study sample and the generalizability of the findings. The study population consisted of patients attending scheduled examinations at an outpatient clinic between 2018 and 2021. While all participants reported prior asbestos exposure, the likelihood and severity of asbestos-related changes varied between groups. Some patients attended due to an officially recognized occupational disease involving asbestos-related pleural or pulmonary changes, whereas others were seen for follow-up occupational health assessments without any known pathological findings. Consequently, the study sample may reflect a selective patient population. Nonetheless, including these diverse groups was essential to ensure the representation of both patients without asbestos-related pleural or lung alterations and those with asbestosis. Well-designed, adequately powered longitudinal studies with robust confounder adjustment are needed to clarify the relationship between asbestosis and impairments in diffusing capacity. Despite the limited availability of research on DLNO in asbestos-related diffusion capacity impairment, this study offers important insights and makes a valuable contribution to the existing body of literature.

To the best of our knowledge, this was the first study to compare low-dose high resolution computed tomography, DLCO and DLNO in asbestos-exposed workers. Although tendencies towards more sensitive detection of diffusion capacity impairment were observed for DLNO over DLCO, DLNO was not clearly superior to DLCO regarding detection and severity assessment of asbestosis. Furthermore, DLNO was susceptible for interference due to emphysema. Based on our findings and considering the lack of availability of DLNO in clinical routine, DLNO does not seem to have added value for clinical assessment of formerly asbestos exposed workers. DLCO and DLCO/VA, which should be included routinely, correlated with severity of asbestosis. Future studies should investigate potential benefits of including healthy controls and confounders such as emphysema and smoking to further investigate differences of DLNO and DLCO regarding detection of asbestos-related alveolar cell and basal membrane thickening.

## Methods

### Study design and patient selection

This is a cross-sectional study of patients visiting the outpatient clinic of the Institute of Occupational, Social and Environmental Medicine, RWTH Aachen University Hospital between September 2018 and February 2021. All patients underwent a standardized medical history, physical examination, pulmonary function testing, DLCO and DLNO, and low-dose high resolution computed tomography. DLNO was incorporated into clinical routine in July 2018 at the outpatient clinic, and all patients visiting from that point onward underwent routine DLNO measurements.

Inclusion criterion was a visit in the outpatient clinic between September 1st 2018 and February 28th 2021 with documented former asbestos exposure. Patients were selected from three groups: (1) acknowledged occupational disease with or without compensation due to asbestos-associated pleural thickening or asbestosis, (2) assessment for occupational disease or (3) participation in a surveillance program for formerly asbestos exposed workers, either Asbestos Surveillance Program Aachen (ASPA)^13–15^ or “EVA Lunge”^16^. All patients included in the study reported a history of asbestos exposure. Patients with recognized occupational diseases presented with pleural thickening and/or asbestosis, as the presence of at least one of these conditions is a prerequisite for official recognition. For those undergoing assessment for a potential occupational disease, referral by other physicians had suggested the possibility of such a diagnosis, though it had not been confirmed. A total of 190 patients (51.2%) presented with either confirmed or suspected occupational disease, while 181 patients (48.8%) were enrolled in surveillance programs. Participants in these surveillance programs also reported asbestos exposure but exhibited no asbestos-related pleural or pulmonary changes. Consequently, the likelihood and severity of asbestos-related changes varied significantly between the groups. Initially, 519 patients were included. For patients with multiple visits, the first was selected. Thus, the analysis presented herein includes data of the first visit of patients at the outpatient clinic between September 2018 and February 2021. Exclusion criteria were a suspected or confirmed diagnosis of lung cancer, mesothelioma, other cancers or collagen disease at the time of the visit, or refusal of pulmonary function testing, DL or low-dose high resolution computed tomography at that date. No female patients visited the outpatient clinic with former asbestos exposure during the reported time period. After removal of patients with additional visits and application of the exclusion criteria, 371 patients were included in data analysis.

The study was conducted in accordance with the Declaration of Helsinki and approved by the Ethics Committee at the RWTH Aachen Faculty of Medicine (EK 094 − 21). Informed consent to participate was waived by the Ethics Committee at the RWTH Aachen Faculty of Medicine for analysis of anonymous clinical routine data.

### Group selection

Group selection was based solely on low-dose high resolution computed tomography findings. Patients with no pleural findings, asbestosis or emphysema were put into the group “no findings”. Patients who showed pleural findings without asbestosis or emphysema were combined in a subgroup labelled “only pleural findings ”. Asbestosis patients were divided into those with a score of irregular and/or linear opacities of 2–3 or at least 4; since pleural findings and asbestosis frequently coincide, additional pleural findings did not result in an exclusion from this group. “Only emphysema” contains patients with emphysema without pleural findings or irregular and/or linear opacities. In “mixed findings” all patients with at least two of pleural findings, asbestosis and emphysema, that were not in the asbestosis groups, were included.

### Pulmonary function test

All pulmonary function testing were performed using a commercial device (Vyaire medical, MasterScreen, Hoechberg, Germany). Spirometric data were derived from a maneuver sequence incorporating a slow deep exhalation, maximal inhalation and forced exhalation adhering to ERS technical standards for standardization of spirometry^49^ and technical standards on interpretive strategies^50^. Forced vital capacity (FVC), forced expiratory volume in 1 s (FEV1) and FEV1/FVC ratio were recorded. Data were normalized based on the reference values provided by the Global Lung Function Initiative (GLI) 2012 reference equations^51^ for spirometry.

### Lung diffusion capacity (DL) – DLCO and DLNO

All DL were performed using a commercial device (Vyaire medical, MasterScreen, Hoechberg, Germany). Single breath diffusion capacity measurement was performed according to technical standards of European Respiratory Society for CO^52^ and NO^53^.

Placed in upright seating position with minimally extended neck, nose clip and lips closed around the mouthpiece of the test device, DL data were derived from a maneuver sequence incorporating a complete exhalation, maximal inhalation, a breath holding period of 10 s for CO and 6 s for NO and maximum exhalation. After a 4-minute pause in seated position the maneuver was repeated. DL was performed twice for CO and twice for NO. DL was only considered if the inspiratory volume of the maneuvers was at least 85% of vital capacity determined with spirometry.

DLCO data were normalized using the reference equations of the Global Lung Function Initiative (GLI) as official ERS technical standard^54^. For DLNO, we used reference equations by Munkholm and colleagues^55^ because those reference equations were established on the MasterScreen™ device that was used in our study^56,57^.

Haemoglobin (Hb) values were determined in all patients per capillary blood gas analysis by a commercial device (Eschweiler GmbH & Co. KG, Kiel, Germany) and DLCO was corrected according to MacIntyre et al.^21^.

### Low dose high resolution computed tomography

Low-dose high resolution computed tomography was performed in the Department of Diagnostic and Interventional Radiology of the chest during inspiratory breath hold. Imaging was conducted with the technical standards with uniform acquisition and reconstruction parameters of the Working Group for Diagnostic Radiology in Occupational and Environmental Diseases (AG DrauE) of the German Roentgen Society^58^. Assessment using the ICOERD^8,9^ as standard for pneumoconiosis assessment^59^ was performed for each patient. The International Classification of Occupational and Environmental Respiratory Diseases (ICOERD) grading system provides a standardized method designed to evaluate and quantify parenchymal and pleural abnormalities observed on high-resolution computed tomography^60^. It was developed for individuals with suspected or confirmed occupational or environmental exposure to harmful dusts and agents to detect and classify pneumoconiosis, such as silicosis, coal workers’ pneumoconiosis, and asbestosis. The grading system provides a structured scoring system that grades various radiological findings, such as irregular and linear opacities, ground-glass, emphysema, and honeycombing—within six anatomical lung zones (upper, middle, lower; right and left), assigning severity scores from 0 (no abnormality) to 3 (severe abnormality) for each zone. Large opacities are recorded separately. The cumulative scores for each parenchymal abnormality assist in evaluating disease extent and severity, improving diagnostic consistency, and supporting epidemiological studies or medico-legal evaluations, such as in occupational disease recognition procedures. Pleural changes, including pleural thickening and calcification, are evaluated distinctly. This comprehensive approach allows for a more nuanced characterization of occupational lung diseases, offering superior sensitivity and specificity compared to chest radiographs, particularly in early or subtle cases of pneumoconiosis. In this study, parenchymal changes of the lungs were assessed for rounded opacities, irregular and/or linear opacities, ground glass, honeycombing, consolidations, and emphysema formations. Following the German guidelines for diagnosis of asbestosis as occupational disease^11^ asbestosis was assumed with a score of ≥ 4 bilateral irregular opacities in the lower lung fields. According to Helsinki Criteria^6^, asbestosis is indicated by bilateral irregular and/or linear opacities with a score ≥ 2 or bilateral honeycombing with a score ≥ 2. For pleural changes visceral and parietal findings, as well as pleural mediastinal or diaphragmal changes were distinguished and classified depending on location, extent, and thickness. Additionally, pleural calcifications were registered. For this study, emphysema was diagnosed solely according to low-dose high resolution computed tomography findings if emphysema formation with a sum grade ≥ 1 was observed according to ICOERD^8,9^.

All low-dose high resolution computed tomography were evaluated by one of two occupational physicians as A-reader and always the same experienced occupational physician as B-reader. Additionally, a radiology findings report was generated by a radiologist.

For Receiver-operating-characteristics (ROC-) curve analysis the diagnosis of asbestosis was determined based on CT findings in accordance with the German guidelines for the diagnosis of asbestosis as an occupational disease^11^. The presence of a score of ≥ 4 bilateral irregular opacities in the lower lung fields was assumed to be asbestosis.

The diagnosis of asbestosis is typically characterised by a history of sufficient asbestos exposure, a latency period of at least 10 years, imaging findings consistent with pulmonary fibrosis, and the exclusion of other potential causes of interstitial lung disease^11,61^. In the present study, exposure and latency were given since patients were only assigned to the outpatient clinic if both aspects were assessed and positive. If alternative etiologies for pulmonary fibrosis were to be considered, these would have been noted in the corresponding occupational disease procedure. This was not the case for patients included in this study. Thus, we concluded that all 22 patients had a sufficient diagnosis of asbestosis.

### Statistical analysis

Data analysis was performed using SPSS Version 29.0.0.0 (Statistical Package for the Social Sciences, Inc., Chicago, IL, USA) and Graph Pad Prism 10.1.2 (GraphPad, La Jolla, CA). Data are shown as median and range or number and percentages (Tables 1, 2 and 3), median with minimum to maximum (box plot, Fig. 1) or mean ± SD (Fig. 3) and n indicates the number of patients. In Table 1, missing data is indicated by the number of patients included. For all other tables and figures no missing data were present since the existence of those values was an inclusion criterion.

Normal distribution was assessed by QQ plots. Data analysis of comparison of groups was performed via one-way ANOVA with post hoc Dunnett’s test (Table 2 + 3 and Fig. 1). For identification of association of DLCO and DLNO (in %pred) with smoking (number of pack years) adjusted for asbestos exposure (fibre years) and CT findings (score of irregular opacities, emphysema and pleural changes according to ICOERD) multivariable linear regression models were computed (Table 4). Asbestos exposure and asbestos associated morphological changes of the lungs and pleura were included due to reported associations with reductions in DL.

ROC curve analysis was conducted and optimal cut off was identified with Youden index to determine sensitivity and specificity (Table 5; Fig. 2). To account for possible influences of emphysema on the relation of DL and score of irregular opacities, we conducted a sensitivity analysis excluding patients with low-dose high resolution computed tomography detected emphysema (Fig. 3A + B). Pearson correlation (*ρ)* was performed for all (*n* = 371) patients and after exclusion of patients who had an emphysema score ≥ 1 (*n* = 224) with depiction of simple linear regression. Comparison of *ρ* was conducted after exclusion of patients with emphysema^62^ (Fig. 3).

Differences between groups were assumed to be significant with *p* < 0.05.

## Data Availability

The datasets used and/or analysed during the current study are available from the corresponding author on reasonable request.

## References

[1] DGUV publications. DGUV Statistics 2021 Figures and long-term trends. Available at https://publikationen.dguv.de/zahlen-fakten/ueberblick/4593/dguv-statistics-2021-figures-and-long-term-trends

[2] Shi, P. et al. Trends in global, regional and National incidence of pneumoconiosis caused by different aetiologies: An analysis from the global burden of disease study 2017. *Occup. Environ. Med.* **77**, 407–414 (2020). 32188634 10.1136/oemed-2019-106321

[3] Bianchi, C. et al. Latency periods in asbestos-related mesothelioma of the pleura. *Eur. J. cancer Prevention: Official J. Eur. Cancer Prev. Organisation (ECP)*. **6**, 162–166 (1997). 9237066

[4] Federal Institute for Occupational Safety and Health. BAuA - Occupational Diseases. Available at https://www.baua.de/EN/Service/Legislative-texts-and-technical-rules/Occupational-diseases/Occupational-diseases_node.html

[5] Kishimoto, T. et al. Clinical, radiological, and pathological investigation of asbestosis. *Int. J. Environ. Res. Public Health*. **8**, 899–912 (2011). 21556185 10.3390/ijerph8030899 PMC3083676

[6] Kawabata, Y. et al. Asbestos exposure increases the incidence of histologically confirmed usual interstitial pneumonia. *Histopathology* **68**, 339–346 (2016). 26046696 10.1111/his.12751

[7] Attanoos, R. L., Alchami, F. S., Pooley, F. D. & Gibbs, A. R. Usual interstitial pneumonia in asbestos-exposed cohorts - concurrent idiopathic pulmonary fibrosis or atypical asbestosis? *Histopathology* **69**, 492–498 (2016). 26864248 10.1111/his.12951

[8] Hering, K. G., Hofmann-Preiß, K. & Kraus, T. [Update: standardized CT/HRCT classification of occupational and environmental thoracic diseases in Germany]. [German]. *Der Radiologe*. **54**, 363–384 (2014). 24737105 10.1007/s00117-014-2674-y

[9] Suganuma, N. et al. Reliability of the proposed international classification of high-resolution computed tomography for occupational and environmental respiratory diseases. *J. Occup. Health*. **51**, 210–222 (2009). 19372629 10.1539/joh.l8030

[10] Vainio, H., Oksa, P., Tuomi, T., Vehmas, T. & Wolff, H. Helsinki criteria update 2014: Asbestos continues to be a challenge for disease prevention and attribution. *Epidemiol. Prev.* **40**, 15–19 (2016). 26951728 10.19191/EP16.1S1.P015.025

[11] Kraus, T. & Teschler, H. Update of the AWMF S2k guideline diagnostics and assessment of occupational Asbestos-Related Diseases - What’s new?? *Pneumologie (Stuttgart Germany)*. **75**, 201–205 (2021). 33728629 10.1055/a-1350-1078

[12] Rehbock, B., Hofmann-Preiß, K. & Kraus, T. [Pitfalls in diagnostic imaging and assessment of benign asbestos-related thoracic diseases]. [German]. *RoFo: Fortschr. Auf Dem Gebiete Der Rontgenstrahlen Und Der Nuklearmedizin*. **184**, 412–419 (2012). 10.1055/s-0031-1299055 22549551

[13] Das, M. et al. Asbestos surveillance program Aachen (ASPA): initial results from baseline screening for lung cancer in asbestos-exposed high-risk individuals using low-dose multidetector-row CT. *Eur. Radiol.* **17**, 1193–1199 (2007). 17047960 10.1007/s00330-006-0426-8

[14] Eisenhawer, C., Felten, M. K., Tamm, M., Das, M. & Kraus, T. Radiological surveillance of formerly asbestos-exposed power industry workers: rates and risk factors of benign changes on chest X-ray and MDCT. *J. Occup. Med. Toxicol. (London England)*. **9**, 18 (2014). 10.1186/1745-6673-9-18 PMC4012178 24808921

[15] Felten, M. K. et al. Retrospective exposure assessment to airborne asbestos among power industry workers. *J. Occup. Med. Toxicol. (London England)*. **5**, 15 (2010). 10.1186/1745-6673-5-15 PMC2901364 20579364

[16] GVS - Gesundheitsvorsorge. Occupational health screening services for the early detection of lung cancer. [German]. Available at https://gvs.bgetem.de/erweitertes-vorsorgeangebot-zur-frueherkennung-von-lungenkrebs

[17] Kopylev, L., Christensen, K. Y., Brown, J. S. & Cooper, G. S. A systematic review of the association between pleural plaques and changes in lung function. *Occup. Environ. Med.* **72**, 606–614 (2015). 25504898 10.1136/oemed-2014-102468 PMC4687690

[18] Caceres, J. D. & Venkata, A. N. Asbestos-associated pulmonary disease. *Curr. Opin. Pulm. Med.* **29**, 76–82 (2023). 36630203 10.1097/MCP.0000000000000939

[19] Karrasch, S. et al. Acute effects of hypertonic saline inhalation on nitric oxide pulmonary diffusing capacity in healthy adults. *Respir. Physiol. Neurobiol.* **258**, 40–46 (2018). 30261306 10.1016/j.resp.2018.09.007

[20] Plantier, L. et al. Physiology of the lung in idiopathic pulmonary fibrosis. *Eur. Respiratory Review: Official J. Eur. Respiratory Soc.* **27** (2018). 10.1183/16000617.0062-2017 PMC9489199 29367408

[21] Macintyre, N. et al. Standardisation of the single-breath determination of carbon monoxide uptake in the lung. *Eur. Respir. J.* **26**, 720–735 (2005). 16204605 10.1183/09031936.05.00034905

[22] Ströker, L., Peldschus, K., Herold, R., Harth, V. & Preisser, A. M. Restrictions of VC and DLCO in relation to asbestos-related computed tomographic findings quantified by ICOERD-based parameters. *BMC Pulm. Med.* **22**, 236 (2022). 35725440 10.1186/s12890-022-02022-x PMC9208103

[23] Preisser, A. M. et al. Relations between vital capacity, CO diffusion capacity and computed tomographic findings of former asbestos-exposed patients: a cross-sectional study. *J. Occup. Med. Toxicol. (London England)*. **15**, 21 (2020). 10.1186/s12995-020-00272-1 PMC7328276 32625240

[24] Brims, F. et al. Correlation of lung function with ultra-low-dose CT-detected lung parenchymal abnormalities: A cohort study of 1344 asbestos exposed individuals. *BMJ Open. Respiratory Res.* **9** (2022). 10.1136/bmjresp-2022-001366 PMC9806062 36581353

[25] Abejie, B. A., Wang, X., Kales, S. N. & Christiani, D. C. Patterns of pulmonary dysfunction in asbestos workers: A cross-sectional study. *J. Occup. Med. Toxicol. (London England)*. **5**, 12 (2010). 10.1186/1745-6673-5-12 PMC2890695 20525229

[26] Mouronte-Roibás, C. et al. COPD, emphysema and the onset of lung cancer. A systematic review. *Cancer Lett.* **382**, 240–244 (2016). 27666776 10.1016/j.canlet.2016.09.002

[27] Slatore, C. G., Baumann, C., Pappas, M. & Humphrey, L. L. Smoking behaviors among patients receiving computed tomography for lung cancer screening. Systematic review in support of the U.S. Preventive services task force. *Annals Am. Thorac. Soc.* **11**, 619–627 (2014). 10.1513/AnnalsATS.201312-460OC 24701999

[28] Garcia-Rio, F. et al. Prevalence of reduced lung diffusing capacity and CT scan findings in smokers without airflow limitation: a population-based study. *BMJ Open. Respiratory Res.* 10 (2023). 10.1136/bmjresp-2022-001468 PMC9884864 36707127

[29] Tesser, C. D. Why is quaternary prevention important in prevention? *Rev. Saude Publica*. **51**, 116 (2017). 29211199 10.11606/S1518-8787.2017051000041 PMC5708264

[30] Manners, D. et al. Correlation of ultra-low dose chest CT findings with physiologic measures of asbestosis. *Eur. Radiol.* **27**, 3485–3490 (2017). 28083692 10.1007/s00330-016-4722-7

[31] Sette, A. et al. Thin-section CT abnormalities and pulmonary gas exchange impairment in workers exposed to asbestos. *Radiology* **232**, 66–74 (2004). 15220494 10.1148/radiol.2321030392

[32] Copley, S. J. et al. Asbestos-induced and smoking-related disease: Apportioning pulmonary function deficit by using thin-section CT. *Radiology* **242**, 258–266 (2007). 17090711 10.1148/radiol.2421051167

[33] Zu, K., Tao, G. & Goodman, J. E. Pleural plaques and lung function in the Marysville worker cohort: A re-analysis. *Inhalation Toxicol.* **28**, 514–519 (2016). 10.1080/08958378.2016.1210704 27569523

[34] Miller, A., Warshaw, R. & Nezamis, J. Diffusing capacity and forced vital capacity in 5,003 asbestos-exposed workers: Relationships to interstitial fibrosis (ILO profusion score) and pleural thickening. *Am. J. Ind. Med.* **56**, 1383–1393 (2013). 24038345 10.1002/ajim.22239

[35] Schikowsky, C., Felten, M. K., Eisenhawer, C., Das, M. & Kraus, T. Lung function not affected by asbestos exposure in workers with normal computed tomography scan. *Am. J. Ind. Med.* **60**, 422–431 (2017). 28370144 10.1002/ajim.22717

[36] Baur, X. & Wilken, D. [Effect of asbestos fibre dust exposures on lung function—a systematic review]. [German]. *Pneumologie (Stuttgart Germany)*. **64**, 81–110 (2010). 20143281 10.1055/s-0029-1243815

[37] Barisione, G. et al. Value of lung diffusing capacity for nitric oxide in systemic sclerosis. *Physiological Rep.* **7**, e14149 (2019). 10.14814/phy2.14149 PMC6603284 31264386

[38] Barisione, G., Brusasco, C., Garlaschi, A., Baroffio, M. & Brusasco, V. Lung diffusing capacity for nitric oxide as a marker of fibrotic changes in idiopathic interstitial pneumonias. *J. Appl. Physiol. (Bethesda Md. : 1985)*. **120**, 1029–1038 (2016). 10.1152/japplphysiol.00964.2015 26893034

[39] Agusti, A. G., Roca, J., Rodriguez-Roisin, R., Xaubet, A. & Agusti-Vidal, A. Different patterns of gas exchange response to exercise in asbestosis and idiopathic pulmonary fibrosis. *Eur. Respir. J.* **1**, 510–516 (1988). 3169220

[40] van der Lee, I. et al. Nitric oxide diffusing capacity versus spirometry in the early diagnosis of emphysema in smokers. *Respir. Med.* **103**, 1892–1897 (2009). 19586765 10.1016/j.rmed.2009.06.005

[41] Dressel, H. et al. Lung diffusing capacity for nitric oxide and carbon monoxide in relation to morphological changes as assessed by computed tomography in patients with cystic fibrosis. *BMC Pulm. Med.* **9**, 30 (2009). 19531222 10.1186/1471-2466-9-30 PMC2708126

[42] Winkler, A. et al. Combined diffusing capacity for nitric oxide and carbon monoxide as predictor of bronchiolitis obliterans syndrome following lung transplantation. *Respir. Res.* **19**, 171 (2018). 30200966 10.1186/s12931-018-0881-1 PMC6131787

[43] Huuskonen, O., Kivisaari, L., Zitting, A., Kaleva, S. & Vehmas, T. Emphysema findings associated with heavy asbestos-exposure in high resolution computed tomography of Finnish construction workers. *J. Occup. Health*. **46**, 266–271 (2004). 15308825 10.1539/joh.46.266

[44] Mabila, S. L. et al. Effects of commodity on the risk of emphysema in South African miners. *Int. Arch. Occup. Environ. Health*. **93**, 315–323 (2020). 31701235 10.1007/s00420-019-01483-8

[45] Clark, K. A. et al. Pleural plaques and their effect on lung function in Libby vermiculite miners. *Chest* **146**, 786–794 (2014). 24810738 10.1378/chest.14-0043

[46] Clark, K. A., Flynn, J. J., Karmaus, W. J. J. & Mohr, L. C. The effects of pleural plaques on longitudinal lung function in vermiculite miners of Libby, Montana. *Am. J. Med. Sci.* **353**, 533–542 (2017). 28641716 10.1016/j.amjms.2017.03.033

[47] Miller, A. et al. Libby amphibole disease: pulmonary function and CT abnormalities in vermiculite miners. *J. Occup. Environ. Med.* **60**, 167–173 (2018). 29200190 10.1097/JOM.0000000000001178 PMC5805586

[48] Park, E. K., Yates, D. H. & Wilson, D. Lung function profiles among individuals with nonmalignant Asbestos-related disorders. *Saf. Health Work*. **5**, 234–237 (2014). 25516818 10.1016/j.shaw.2014.07.007 PMC4266811

[49] Graham, B. L. et al. Standardization of spirometry 2019 update. An official American thoracic society and European respiratory society technical statement. *Am. J. Respir. Crit Care Med.* **200**, e70–e88 (2019). 31613151 10.1164/rccm.201908-1590ST PMC6794117

[50] Stanojevic, S. et al. ERS/ATS technical standard on interpretive strategies for routine lung function tests. *Eur. Respir. J.* 60 (2022). 10.1183/13993003.01499-2021 34949706

[51] Quanjer, P. H. et al. Multi-ethnic reference values for spirometry for the 3-95-yr age range: the global lung function 2012 equations. *Eur. Respir. J.* **40**, 1324–1343 (2012). 22743675 10.1183/09031936.00080312 PMC3786581

[52] Graham, B. L. et al. 2017 ERS/ATS standards for single-breath carbon monoxide uptake in the lung. *Eur. Respir. J.* 49 (2017). 10.1183/13993003.00016-2016 28049168

[53] Zavorsky, G. S. et al. Standardisation and application of the single-breath determination of nitric oxide uptake in the lung. *Eur. Respir. J.* 49 (2017). 10.1183/13993003.00962-2016 28179436

[54] Stanojevic, S. et al. Official ERS technical standards: global lung function initiative reference values for the carbon monoxide transfer factor for Caucasians. *Eur. Respir. J.* 50 (2017). 10.1183/13993003.00010-2017 28893868

[55] Munkholm, M. et al. Reference equations for pulmonary diffusing capacity of carbon monoxide and nitric oxide in adult Caucasians. *Eur. Respir. J.* 52 (2018). 10.1183/13993003.00677-2015 29903858

[56] Radtke, T. et al. Lung diffusing capacity for nitric oxide measured by two commercial devices: A randomised crossover comparison in healthy adults. *ERJ Open. Res.* 7 (2021). 10.1183/23120541.00193-2021 PMC8381155 34435029

[57] Radtke, T., Hua-Huy, T., Dressel, H. & Dinh-Xuan, A. T. Of the need to reconcile discrepancies between two different reference equations for combined single-breath DLNO-DLCO in systemic sclerosis. *Eur. Respir. J.* 53 (2019). 10.1183/13993003.02109-2018 30655283

[58] Nagel, H. et al. Protocol recommendations of the AG DRauE for conducting low-dose volume HRCT examinations of the lungs. [German]. *Fortschr Röntgenstr* ; 189: 553–575. (2017).

[59] Tamura, T. et al. Relationships (I) of international classification of High-resolution computed tomography for occupational and environmental respiratory diseases with the ILO international classification of radiographs of pneumoconioses for parenchymal abnormalities. *Ind. Health*. **53**, 260–270 (2015). 25810444 10.2486/indhealth.2014-0073 PMC4463184

[60] Kusaka, Y., Hering, K. G. & Parker, J. E. *International Classification of HRCT for Occupational and Environmental Respiratory Diseases* (Springer, 2005).

[61] Official statement of the American Thoracic Society. Diagnosis and initial management of nonmalignant diseases related to asbestos. *Am. J. Respir. Crit Care Med.* **170**, 691–715 (2004). 15355871 10.1164/rccm.200310-1436ST

[62] Lehnhard, W. & Lehnhard, A. Testing the Significance of Correlations (2014).

